# A practical guide to prescribing sublingual immunotherapy tablets in North America for pediatric allergic rhinoconjunctivitis: an injection-free allergy immunotherapy option

**DOI:** 10.3389/fped.2023.1244146

**Published:** 2023-10-04

**Authors:** Michael Blaiss, Lawrence DuBuske, Hendrik Nolte, Morten Opstrup, Karen Rance

**Affiliations:** ^1^ Department of Pediatrics, Medical College of Georgia, Augusta, GA, United States; ^2^ Department of Medicine, The George Washington University Hospital, Washington, DC, United States; ^3^ ALK, Bedminster, NJ, United States; ^4^ ALK-Abelló A/S, Hørsholm, Denmark

**Keywords:** allergic rhinitis, allergen immunotherapy, children, checklist, decision tree, management, sublingual

## Abstract

Allergic rhinoconjunctivitis (ARC) is a common disease that affects individuals of all ages. Pediatricians may be the first (and only) point of care for children with ARC. Sublingual immunotherapy (SLIT)-tablets are a convenient at-home, injection-free allergy immunotherapy option that can be used for the treatment of ARC. This paper provides a practical guide for pediatricians to aid in prescribing SLIT-tablets to children with ARC in North America. Topics include a summary of the available SLIT-tablets and their efficacy and safety, guidance on when SLIT-tablets are an appropriate option, and how to diagnose ARC and identify culprit allergens. Practical guidance is also provided through a proposed decision tree, a prescribing checklist and prescribing procedures, and suggested follow-up assessments.

## 1. Introduction

Allergic rhinoconjunctivitis (ARC) is a common disease that affects individuals of all ages around the world. The most common symptoms of ARC are runny nose, congestion, sneezing, and itchy, watery eyes. At times, the symptoms of ARC can be severe, disrupting sleep, interfering with the ability to perform daily activities, and inducing anxiety, irritability, fatigue, and frustration (1–4). The symptoms of ARC can even impair cognitive function and affect school performance (2, 5). High pollen levels or the presence of ARC symptoms are associated with poorer performance by adolescents on school exams (6, 7). Children with ARC also often have asthma and atopic dermatitis and are prone to ear infections and sinusitis, which may contribute to the burden of disease (2, 8, 9).

The symptoms of ARC are manifestations of an IgE-mediated response to seasonal allergens such as grass, ragweed, or tree pollen or perennial allergens such as house dust mites (HDM), molds, or animal dander. Pharmacotherapy options for ARC typically treat symptoms by targeting the histamine and other inflammatory mediators that are released or elevated after the IgE response is triggered by an allergen. These symptom-relieving medications include antihistamines, intranasal corticosteroids, and leukotriene inhibitors. Allergen immunotherapy (AIT) is another treatment option for ARC that effectively reduces symptoms by modifying the disease mechanisms that drive ARC (10). Three years of AIT is generally recommended to achieve a long-term sustained effect after treatment has been stopped (11, 12). Subcutaneous immunotherapy (SCIT; aka “allergy shots”) has been practiced for over 100 years and is the primary means of AIT used in the US (13, 14). Administration of SCIT is recommended in a clinical setting typically every 1–6 weeks with a 30 min wait period after the injection to monitor for severe systemic allergic reactions (15). Thus, the inconvenience and time needed for the frequent clinic visits are common reasons why patients decline SCIT or start SCIT but then stop (14). In addition, fear of needles or the prospect of repeated injections may make SCIT particularly unappealing for children. These factors likely contribute to the underutilization of SCIT. Many patients with ARC are simply unaware of AIT as a treatment option (3, 16).

Sublingual immunotherapy (SLIT) is an injection-free form of AIT administered by drops or tablets with a safety profile that allows for at-home administration. Only the first dose needs to be administered in a clinical setting to ensure the treatment is tolerated (17). The convenience and injection-free nature of SLIT may be an appealing AIT option for children (18, 19). SLIT-drops are an off-label use of SCIT extracts in the US. There are little data on the efficacy and safety of SLIT-drops in North America, and they are not FDA approved or endorsed by the American Academy of Allergy, Asthma & Immunology (AAAAI) or the American College of Allergy, Asthma & Immunology (ACAAI) (17). In addition, development of eosinophilic esophagitis may be more likely with SLIT-drop use of North American SCIT extracts than tablets because of differences in contact of the released allergen with the lower esophagus (20). SLIT-tablets to treat grass, ragweed, and HDM-associated ARC are FDA approved in the US and a tree SLIT-tablet is also approved in Canada (Table 1). This paper provides a practical guide for pediatricians to aid in prescribing SLIT-tablets to children with ARC. The information presented is based on the authors’ clinical experience and non-systematic literature searches.

**Table 1:** Indications for SLIT-tablets approved in North America.

SLIT-tablet allergen	Brand name	Country approval	Date initially approved	Indicated age	Dosing regimen
5-grass mixture	Oralair®	Canada	2016	5–50 years	Daily pre-seasonally (4 months before the pollen season) and co-seasonally
		US	2014	5–65 years	
Timothy grass	Grastek®	Canada	2013	≥5 years	Daily pre-seasonally (at least 8 weeks before the pollen season) and co-seasonally
		US	2014	5–65 years	Daily pre-seasonally (at least 12 weeks before the pollen season) and co-seasonally
Short ragweed	Ragwitek®	Canada	2013	≥5 years	Daily pre-seasonally (at least 12 weeks before the pollen season) and co-seasonally
		US	2013	5–65 years	
Tree (Birch)	Itulatek^™^	Canada	2019	18–65 years	Daily pre-seasonally (at least 16 weeks before the pollen season) and co-seasonally
House dust mite	Acarizax®	Canada	2017	12–65 years	Daily year-round
	Odactra®	US	2017	12–65 years	

SLIT, sublingual immunotherapy.

## 2. Role of pediatricians in ARC and AIT

An international survey found that the self-reported prevalence of ARC symptoms in the previous year was 13.3% for adolescents ages 13–14 years and 7.7% for children ages 6–7 years (1). Because of this high prevalence of ARC in children and an insufficient number of allergy specialists, primary care providers fulfill a critical need in the management of ARC (21). In a nationwide survey of individuals in the US who had ever been diagnosed with ARC, 41% of children were diagnosed by a pediatrician and 22% by a family medicine practitioner (22). Furthermore, the majority of patients reported seeking allergy care from a primary care provider rather than an allergist (22). Despite the importance of primary care providers in the management of ARC, surveys have identified that primary care providers may not feel adequate in understanding AIT, a critical aspect of ARC care. A survey of primary care providers across 5 countries in Europe found that only 10%–29% perceived their confidence level of AIT as “adequate” (23), although 37% of the pediatricians responding to the survey perceived themselves as having adequate knowledge of AIT (24). Thus, SCIT is generally prescribed by allergists or otolaryngologists because it requires knowledge of oftentimes complex extract preparation, space and personnel to prepare extracts, reimbursement procedures, clinic resources and procedures to manage potential anaphylactic reactions, and time, space, and administrative constraints to manage the recurring visits (15, 16, 25, 26). SLIT-tablets have simple daily dosing and no need for frequent clinic visits, which reduces the complexity and logistics that are associated with SCIT. As such, SLIT-tablets can be prescribed and managed in a primary care office provided the physician has experience in the assessment and management of allergic diseases (17).

## 3. SLIT-tablet efficacy and safety in children

There are currently 5 SLIT-tablets approved for the treatment of ARC in North America; 4 of the SLIT-tablets are approved for children or adolescents (Table 1). The ability of the SLIT-tablets to significantly reduce ARC symptoms and symptom-relieving medication use in children or adolescents has been demonstrated in randomized, double-blind, placebo-controlled trials (27–32). AIT may also have the added benefit of preventing progression to asthma in patients with ARC. In a 5-year prospective trial, children with ARC and no history of asthma who received the timothy grass SLIT-tablet for 3 years had a significantly lower risk of experiencing asthma symptoms or using asthma medication at year 5 compared with those who received placebo (odds ratio = 0.66, *p* = 0.04) (33). Real-world retrospective studies have also found a lower percentage of patients receiving timothy grass or 5-grass SLIT-tablets developed asthma compared with untreated patients (34, 35). Similar studies have yet to be conducted for the other SLIT-tablets. An additional potential benefit of AIT is prevention of new allergen sensitizations, although more data are needed on this topic (36).

Anaphylaxis is a possibility with AIT in general since treatment exposes a patient to the allergen to which they are allergic, however, anaphylaxis is rare with SLIT. Across the entire clinical development program of the timothy grass, ragweed, tree, and HDM SLIT-tablets comprising over 14,000 subjects, the anaphylaxis rate was 0.03% with active treatment (compared with 0.02% with placebo) (37). By comparison, the rate of anaphylaxis to penicillin is estimated to be between 0.015% to 0.04% (38). There have been no reported deaths related to SLIT-tablets.

The most common adverse events with SLIT-tablets are local allergic reactions that occur at or near the site of tablet administration (e.g., throat irritation, oral pruritus, ear pruritus; Table 2) (39, 40). These reactions are expected since the patient is being exposed to the allergen that causes their allergic symptoms. The vast majority of the adverse events related to SLIT-tablets are mild or moderate; in a pooled analysis of pediatric data from timothy grass SLIT-tablet trials, only 3% of adverse events related to the grass SLIT-tablet were severe (41). Severe local allergic reactions are not a major safety issue unless they compromise the airway. To date, there have been no such events reported in SLIT-tablet clinical trials (38, 40). However, the local allergic reactions are a tolerance issue that can be bothersome for patients and lead to treatment discontinuation. Data from clinical trials show that the local allergic reactions typically resolve within 30–60 min after SLIT-tablet administration and recur for less than 2 weeks (39). Caregivers considering SLIT-tablets for their child need to be informed during the shared decision making process about the potential local allergic reactions that can occur with the SLIT-tablets, the expected duration of the reactions, and guidance on how to manage them to help children maintain treatment. This information is important to encourage adherence.

**Table 2:** Most common side effects that occur with SLIT-tablets (39, 40).

• Throat irritation • Oral pruritus • Ear pruritus • Mouth edema	• Oral paresthesia • Lip swelling • Swollen tongue

SLIT, sublingual immunotherapy.

## 4. When is SLIT-tablet an appropriate option?

According to SLIT guidelines from the World Allergy Organization, patients with a history of ARC symptoms related to allergen exposure and with documented allergen-specific IgE (sIgE) are eligible for SLIT (Table 3) (19). The FDA prescribing information for the SLIT-tablets indicate that they should be used for the treatment of ARC confirmed by positive skin test or *in vitro* testing for sIgE antibodies (42–45). Despite intensive research efforts, no validated biomarkers have been identified that can predict which patients will successfully respond to AIT. Essentially, SLIT-tablet treatment can be considered for any child (within the approved age range) with a positive sIgE and a clinical history that suggests ARC relevant to the allergen. SLIT-tablets do not need to be reserved only for children whose symptoms are not well-controlled by symptom-relieving medication or who have undesirable side effects to pharmacotherapies, although greater consideration may be given for children with these particular situations (Table 3) (19). In addition, children who are at high risk for developing asthma (i.e., atopic parents; obesity; past history of food allergy or atopic dermatitis) (46, 47) may be good candidates for AIT because of its disease-modifying aspects. There are some children for whom SLIT-tablets may not be a good option, including those who are taking certain medications that may enhance the likelihood or interfere with treatment of a severe allergic reaction to the SLIT-tablets (e.g., antidepressants), or those who are receiving other AIT (Table 3). Some patients and caregivers may choose not to start SLIT-tablets since they require adherence to daily dosing for three years.

**Table 3:** Eligibility criteria for treatment of ARC with SLIT-tablets and FDA-label contraindications (19, 42–45).

General eligibility criteria	Situations for particular consideration of SLIT-tablets	FDA-label contraindications	Situations in which SLIT-tablets may not be optimal
• Clinically relevant history related to allergen exposure • Allergen sensitivity confirmed by skin test or presence of serum allergen-specific IgE upon *in vitro* testing	• Patients whose ARC is uncontrolled with optimal symptom-relieving medications • Patients with undesirable side effects to symptom-relieving medications • Patients who refuse injections • Patients who do not want to be on constant or long-term symptom-relieving medications	• Severe, unstable, or uncontrolled asthma • History of any severe systemic allergic reaction or any severe local reaction to SLIT • A history of eosinophilic esophagitis • Hypersensitivity to any of the inactive ingredients contained in the SLIT-tablets	• Patients with lung disease (e.g., COPD) • Patient has heart disease, irregular heart beat, or hypertension that is not well controlled • Patient is pregnant • Patient or caregiver is unwilling to administer autoinjectable epinephrine in the case of an emergency • Patient is receiving other AIT • Patient is taking medication that may enhance likelihood of severe allergic reactions or interfere with treatment of severe allergic reaction (i.e., beta-blockers, alpha-blockers, cardiac glycosides, diuretics, diphenhydramine, ergot alkaloids, MAOI or tricyclic antidepressants, thyroid hormone)

ARC, allergic rhinoconjunctivitis; FDA, US Food and Drug Administration; MAOI, monoamine oxidase inhibitors; SLIT, sublingual immunotherapy.

## 5. Diagnosis of ARC and identification of culprit allergens

Diagnosis of ARC is determined from clinical history, physical examination, and serum sIgE or skin testing. Detailed guidance on AR diagnosis has been provided by an ACAAI/AAAAI Joint Task Force (48), and simplified guidance pathways for the diagnosis of ARC in primary care have been published (49). Basic guidance to help determine if a child's rhinitis symptoms are attributed to ARC instead of a viral cold is shown in Table 4 (50). In addition, Table 5 provides a list of simple and quick questions that link exposure to prominent, easily remembered US holidays that can help determine clinically relevant allergens associated with symptoms. However, pollen seasons correspond to seasonal temperature changes and therefore vary by geographic location. Pediatricians should verify the typical pollen times in their region. To determine the presence of serum sIgE antibodies toward a suspected culprit allergen that can be treated with the SLIT-tablets available in the US, a limited diagnostic panel is available with a US LabCorp procedure code of 607706. The panel measures sIgE towards the HDM allergens Der *p* and Der f, the grass allergen Phl *p* 5, and the short ragweed allergen Amb a 1. If the results do not indicate positive sIgE to the 4 allergens but the child remains symptomatic, referral to an allergist is recommended.

**Table 4:** Clues to an ARC diagnosis.

Rhinitis may be allergic if: • The eyes are involved • Itching is noticeable—child gives allergic salute, has allergic crease • Exposure to a known allergen reliably causes symptoms • Personal or family history of other allergic diseases • Some children present with a comorbidity (asthma, atopic eczema, rhinosinusitis, hearing difficulties, sleep disturbance, behavior problems, pollen food syndrome). Always ask about nasal symptoms in such patients • Always ask about asthma in children with rhinitis and vice-versa

ARC, allergic rhinoconjunctivitis.

Rhinitis symptoms are nasal running, nasal blockage, itching, sneezing, all of which are common in children due to viral colds. Reproduced from Scadding et al, 2021 (50) under Creative Commons Attribution License.

**Table 5:** Caregiver questions to help determine a child's clinically relevant allergy^a^.

Do ARC symptoms occur	Typical allergen related to symptoms
Between Easter/Passover and Memorial Day	Tree pollen
From Memorial Day to July 4th	Grass pollen
Before and after Labor Day until first frost	Ragweed pollen
When exposed to someone vacuuming a carpet or making a bed	House dust mite
When exposed to a particular animal	Animal dander
When going into a damp basement or immediately after rain fall when outdoors	Molds
When mulching leaves in the fall	Molds

ARC, allergic rhinoconjunctivitis.

^a^ Pollen seasons correspond to seasonal temperature changes and therefore vary by geographic location.

Questions are designed to coincide with seasonal allergen seasons in the US or perennial allergens.

## 6. Decision tree for SLIT-tablets

**Figure 1:**
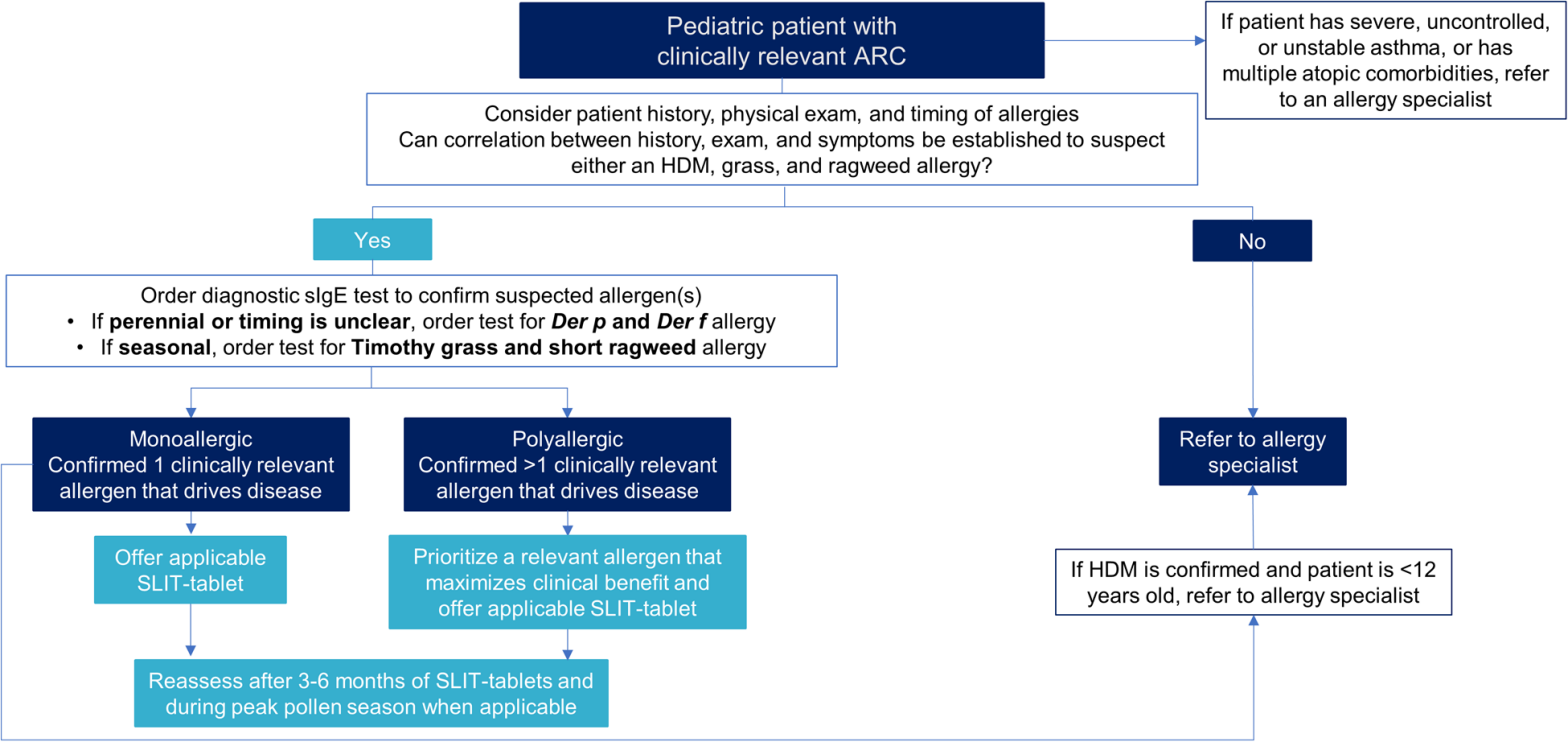
Decision tree for pediatricians between offering SLIT-tablets or referring to an allergy specialist. ARC, allergic rhinoconjunctivitis; Der f, *Dermatophagoides farinae*; Der p, *Dermatophagoides pteronyssinus*; HDM, house dust mite; sIgE, allergen-specific immunoglobulin E; SLIT, sublingual immunotherapy.

Figure 1 is a decision tree to help pediatricians decide between offering SLIT-tablets to their patient or referring the patient to an allergy specialist. Primarily the decision comes down to the asthma status of the patient, whether the patient is monosensitized or polysensitized, and the availability of a SLIT-tablet applicable to the patient's allergy. Patients with severe, uncontrolled, or unstable asthma, or multiple atopic comorbidities, should automatically be referred to an allergy specialist. If the patient does not have asthma or has well-controlled asthma, has an established clinically relevant allergy to only one allergen (monoallergic), and a SLIT-tablet is available that is applicable to the patient's allergy, the SLIT-tablet can be offered. Positive sIgE to multiple allergens may or may not indicate polyallergy and depends on a corresponding clinical history. For example, a patient may have a positive sIgE for ragweed and grass but only have symptoms during ragweed season. Such a patient would be considered polysensitized, but monoallergic, and ragweed SLIT-tablet alone would be appropriate.

The decision to offer SLIT-tablets to patients who are polyallergic is more complicated. Polyallergy is quite common at approximately 50% in children with ARC (51). Some of the polyallergies are among related highly cross-reactive pollen allergens and can be treated with AIT against one of the allergens within the cross-reactive group. The AAAAI/ACAAI Joint Task Force on AIT and the Canadian Society of Allergy and Clinical Immunology endorse this approach (15, 52). For example, the Northern grasses (timothy, sweet vernal, orchard, perennial rye, and meadow) are highly cross-reactive, and allergy to any of these grasses can be treated with the timothy grass SLIT-tablet. Trees are another example. The tree SLIT-tablet that contains a birch extract is effective in reducing symptoms and symptom-relieving medication use during other tree pollen seasons that are related to birch (e.g., hazel, alder, and oak) (53, 54).

If the patient is polyallergic to unrelated allergens, typical practice in the US by allergy specialists is to administer SCIT with multiple extracts that target several of the clinically relevant allergens. In contrast, the selection of one clinically relevant allergen for SCIT or SLIT is standard practice in Europe and Asia. Similarly, to offer a SLIT-tablet for polyallergic patients, the pediatrician needs to decide if it is possible to prioritize one particular allergen to target. Treating for HDM allergy with or without seasonal polyallergy is the first recommended prioritization, followed by treating the seasonal pollen allergen that would maximize clinical benefit. Coadministration of 2 different SLIT-tablets after sequential initiation periods has been shown to be well-tolerated in randomized, double-blind, placebo-controlled safety studies, but efficacy data with coadministration are not yet available (55, 56).

## 7. Prescribing procedures for SLIT-tablets

Each SLIT-tablet has one daily dose that has been determined to be effective and safe in randomized clinical trials. There is no difference in dosing between adults and children, with the exception of the 5-grass SLIT-tablet that requires the dose to be increased over the first 2 days (100 index of reactivity [IR] on day 1, 2× 100 IR on day 2) in children ages 5–17 years before reaching the maintenance dose of 300 IR on day 3 (45). The HDM SLIT-tablet should be taken daily year-round, and seasonal SLIT-tablets should be started at least 8–16 weeks before the expected start of the pollen season to ensure the onset of effect before the season (Table 1). The timothy grass SLIT-tablet can also be taken daily year-round. Data from SLIT trials indicate that initiation of treatment during the pollen season is well tolerated (57). The first dose of any SLIT-tablet must be administered under the supervision of a physician with experience in the diagnosis and treatment of allergic diseases (42–45). The patient then needs to be observed in the office for 30 min to watch for signs and symptoms of anaphylaxis.

There are some important safety instructions that need to be given to caregivers and patients when prescribing SLIT-tablets, most of which are covered in the FDA-approved patient Medication Guides for each SLIT-tablet that should be given to the caregiver. First, caregivers and patients need to be informed of the signs and symptoms of serious allergic reactions (i.e., dyspnea, throat swelling that causes trouble speaking, breathing, or swallowing, dizziness, etc) and instructed to seek immediate medical care and discontinue therapy should any of these occur. In the US, an epinephrine autoinjector is a required co-prescription for SLIT-tablets. Caregivers and patients need to be instructed regarding the appropriate use of the autoinjector and trained in its use. In addition, caregivers and patients also need to be informed that treatment should be stopped if they experience severe or persistent symptoms of esophagitis, if they have persistent or escalating side effects in the mouth or throat, or if patients with asthma have difficulty breathing or their asthma becomes difficult to control. If the patient disrupts treatment for any of these reasons, they need to contact the prescribing physician. Treatment should also be temporarily stopped in case of oral inflammation or wounds (i.e., dental surgery) to allow the oral cavity to heal. Data with the HDM SLIT-tablet suggest that there is no safety issue if treatment is reinitiated after a treatment interruption of approximately 2 weeks (58). If a dose is accidentally missed, the patient should not take two tablets but should wait and take one tablet at their next usually scheduled time. Caregivers and patients also need to be informed about the most common side effects (Table 2) and how often these may last (30–60 min) and recur (about 2 weeks). Caregivers and patients need to be prepared to expect these local allergic reactions and be informed that by themselves these reactions are not dangerous. Otherwise, they may become nervous and stop treatment.

A checklist of steps for prescribing SLIT-tablets to children with ARC is shown in Table 6.

**Table 6:** Checklist for prescribing SLIT-tablets to children with ARC.

□ The patient/caregiver has been verbally informed about the treatment □ Written information about the treatment has been given to the patient/caregiver □ The patient does not have a history of any severe systemic allergic reaction or any severe local reaction to SLIT □ The patient does not have severe, unstable or uncontrolled asthma □ The patient does not have a history of eosinophilic esophagitis □ The patient does not have hypersensitivity to any of the inactive ingredients contained in the SLIT-tablet □ The patient/caregiver has been informed that in case of oral inflammation or wounds to temporarily stop treatment with the tablet to allow complete healing of the oral cavity □ The patient/caregiver has been informed of the most common side-effects □ The patient/caregiver has been informed of the signs and symptoms of serious allergic reactions and instructed to seek immediate medical care and discontinue therapy should any of these occur □ The patient/caregiver has been informed to stop treatment if: • They experience severe or persistent symptoms of esophagitis • They have persistent or escalating side effects in the mouth or throat • They have asthma and it becomes difficult to control or they have difficulty breathing □ The patient/caregiver has been prescribed, instructed in and trained in using an epinephrine autoinjector □ The patient/caregiver has been informed on what to do if treatment is interrupted □ The first dose of the tablet was administered under the supervision of a physician with experience in the diagnosis and treatment of allergic diseases and the patient was observed in the office for at least 30 min following the initial dose.

ARC, allergic rhinoconjunctivitis; SLIT, sublingual immunotherapy.

## 8. Role of the caregiver in administration and monitoring of SLIT-tablet treatment

For younger children, the caregiver should place the SLIT-tablet under the child's tongue to ensure proper sublingual placement. The SLIT-tablets are manufactured using the same technology as other quick dissolving tablets used sublingually in children as young as 1 month of age (e.g., ondansetron orally disintegrating tablets) and will stick to the wet mucosa and dissolve immediately. The child should not swallow for at least 1 min or eat or drink for 5 min. The appropriate age at which a child can self-administer the SLIT-tablet will vary depending on the child, but whomever administers the SLIT-tablet should wash their hands afterward. The child should be observed by the caregiver for at least 5 min after each administration to watch for signs of SLIT-tablet aspiration or an allergic reaction.

## 9. Follow-up visits

Follow-up visits for children prescribed SLIT-tablets are important to assess efficacy, safety, and adherence. Numerous immunologic changes have been characterized in response to AIT in accordance with the disease modifying activity (59), but monitoring of these changes is not practical in the clinic setting (12). Assessment of efficacy relies on the perception of symptoms by patients and caregivers. Clinical improvement with seasonal SLIT-tablets is expected during the first pollen season if treatment is initiated within the recommended preseasonal time period (60). An onset of effect with the HDM SLIT-tablet can be expected in as early as 8 weeks (60). An improvement in symptoms supports continuation for the full recommended 3-year course. If there is no improvement in symptoms after the first year, there is no indication to continue treatment (15). For pediatricians who wish to take a measurement-based care approach, short and simple tools such as a visual analog scale or the validated Rhinitis Control Assessment Test (61) can be used to evaluate ARC symptom control at each visit to compare with a pretreatment value (Table 7). Digital apps, such as the My Pollen Forecast-Allergies app, allows the patient or caregiver to create a digital diary that tracks improvement over time and may be useful for assessing the efficacy of SLIT-tablet treatment. For children with seasonal allergies, a follow-up visit during peak pollen season may be conducted to assess symptom control when it is most needed.

**Table 7:** Recommended questions to assess efficacy, adherence, and safety of SLIT-tablets at follow-up visits.

Efficacy assessment questions^a^	Adherence and safety assessment questions
During the past week… 1. How often do you have nasal congestion? 5 = Never 4 = Rarely 3 = Sometimes 2 = Often 1 = Extremely often	1. Is your child taking their medication as prescribed?
2. How often did you sneeze? 5 = Never 4 = Rarely 3 = Sometimes 2 = Often 1 = Extremely often	2. Do you have any difficulties remembering to give your child their medication?
3. How often did you have watery eyes? 5 = Never 4 = Rarely 3 = Sometimes 2 = Often 1 = Extremely often	3. Has your child missed any doses of their medication in the past week?
4. To what extent did your nasal or other allergy symptoms interfere with your sleep? 5 = Not at all 4 = A little 3 = Somewhat 2 = A lot 1 = All the time	4. Is your child experiencing any side effects from their medication?
5. How often did you avoid any activities because of your nasal or other allergy symptoms? 5 = Never 4 = Rarely 3 = Sometimes 2 = Often 1 = Extremely often	5. Do you have any concerns or questions about your child's medication?
6. How well were your nasal or other allergy symptoms controlled? 5 = Completely 4 = Very 3 = Somewhat 2 = A little 1 = Not at all	6. Do you understand why your child is taking their medication and what it is supposed to do?
	7. Have you been able to obtain your medication from the pharmacy regularly?
	8. Has your child had any changes in his/her routine or lifestyle that may affect their ability to take their medication?

^a^ Questions are from the validated Rhinitis Control Assessment Test. A score of 21 or less indicates ARC symptoms are uncontrolled. Improvement of 3 points is considered clinically meaningful (61).

As with any medication, adherence to the daily SLIT-tablet treatment is critical for its success. Based on data from a prospective randomized study (62), follow-up visits every 3–6 months are recommended to maximize adherence. During these follow-up visits, the pediatrician can ask the patient or caregiver questions to assess adherence (Table 7). If any issues with adherence are revealed, further conversations should be held to emphasize the importance of adherence and address any barriers or issues that are affecting adherence. The use of digital apps or reminders may be useful for helping patients who have difficulty remembering to take their SLIT-tablet (12). Questions to assess the safety of the SLIT-tablet treatment can also be asked during the follow-up visit (Table 7).

## 10. Discussion

In North America, pediatricians may be the first (and only) point of care for children with ARC. SLIT-tablets are a convenient at-home, injection-free AIT option that can be prescribed for children with ARC. The proposed decision tree and prescribing checklist provide pediatricians with the tools to prescribe SLIT-tablets to children.
